# Optimizing nursing home menus in Norway from a sustainability and nutritional perspective

**DOI:** 10.3389/fnut.2026.1776523

**Published:** 2026-04-16

**Authors:** Gizem Aytekin-Sahin, Omur Sahin, Sigrun Henjum

**Affiliations:** 1 Department of Nutrition and Dietetics, Faculty of Health Sciences, Nuh Naci Yazgan University, Kayseri, Türkiye; 2 Department of Nursing and Health Promotion, Faculty of Health Sciences, Oslo Metropolitan University, Oslo, Norway; 3 Department of Computer Engineering, Faculty of Engineering, Erciyes University, Kayseri, Türkiye; 4 School of Economics, Innovation and Technology, Kristiania University of Applied Sciences, Oslo, Norway

**Keywords:** *EvoMeal*, food services, multi objective optimization, nursing home menus, nutrition, sustainability

## Abstract

**Introduction:**

Transforming food services into a more sustainable and healthier systems has become a crucial necessity today. This transformation plays an even more critical role in institutional settings, particularly for vulnerable populations. In nursing homes, where most older adults’ daily meals are organized and provided, the nutritional adequacy, diversity, and environmental footprint of the menus directly influence residents’ overall health and wellbeing. The current study aimed to comprehensively compare 12-week dinner menus (standard menus) and menus created using *EvoMeal*, an artificial intelligence-based menu planning tool (optimized menus) delivered to nursing homes by a catering company in Eastern Norway in terms of nutrient composition, menu quality, and environmental impact (carbon and water footprints).

**Methods:**

A multi-objective optimization method was used to optimize the menus. Standard dinner menus from the catering company were collected and imported into *EvoMeal.* Existing meal contents were not altered; only menu combinations were rearranged. The menus were evaluated for compliance with the Norwegian Food-Based Dietary Guidelines (FBDG). In addition, menu quality was assessed using Elderly Nutrient Rich Food Index 7.3 (E-NRF 7.3), Dietary Diversity Score (DDS), and environmental sustainability using carbon footprint (kg CO_2_-eq) and water footprint (m^3^/ton).

**Results:**

Optimized menus were found to better comply with the Norwegian FBDG. There were no statistically significant differences between the optimized and standard menus in terms of E-NRF 7.3 and DDS. Optimization of the 4-week standard menus resulted in carbon footprint changes ranging from −36.44 to −42.34%, and water footprint changes from −29.37 to −36.12%. In addition, optimization of the 12-week standard menus resulted in a carbon footprint reduction of −40.00%, and a water footprint reduction of -33.09%.

**Discussion:**

To our knowledge, no study has comprehensively focused on optimizing nursing home menus in terms of nutritional composition, menu quality, and environmental impact. *EvoMeal* offers an effective way to reduce the environmental impact of nursing home menus by optimizing menu combinations without altering meal contents. While standard menus in Norwegian nursing homes exhibit relatively high nutritional quality, an optimization approach may be a viable and cost-effective strategy, particularly for improving environmental sustainability.

## 1 Introduction

Norway, like many other countries, is facing a gradual increase in its older adult population (1, 2), and approximately 38,000 people live in nursing homes, of whom three out of four are 80 years old or older (3). Accordingly, the number of older adults who have lost their ability to care for themselves is also increasing (4), and the need for health, social, and nutritional services for older adults also increases. Therefore, nursing homes have emerged as a necessity of modern life, and their numbers are growing (5). However, several studies have shown that older adults living in nursing homes have worse nutritional status when compared to the community-dwelling (6, 7). Especially protein, vitamin D, calcium, magnesium, zinc, B_6_, and folate deficiency were commonly reported in nursing home residents (8). Some studies have also shown increased malnutrition rates among nursing home residents (6, 9), whereas another has indicated raised overweight or obesity rates (10). While achieving optimal nutritional status is a key goal in institutional food services, it is equally important to recognize that many nursing home residents are in the late stages of life, with multiple comorbidities and limited functional capacity. Evidence indicates that inadequate nutritional status, including protein–energy malnutrition, is associated with poorer quality of life among nursing home residents, underscoring the importance of maintaining sufficient and balanced nutrition (11). At the same time, providing nutritionally balanced and enjoyable meals is essential not only for preventing malnutrition but also for supporting residents’ dignity, autonomy, and social wellbeing (12).

Many factors affect the nutritional status of nursing home residents, including menu quality. However, menu planning is a complex problem, involving many factors. The planned menus are expected to meet the target group’s energy and nutrient requirements, be sufficient in terms of quality and quantity, and contain a wide variety of food groups in sufficient quantities (13). In addition, it is important to integrate the sustainable nutrition dimension, which has become increasingly important in recent years, into food services (14, 15), and nutrition is the most powerful factor for optimizing human health and environmental sustainability (16). However, despite the focus on the impact of diets on individual and planetary health, food services in healthcare settings have received very limited attention to date.

In addition to their nutritional implications, menus in institutional food services can have considerable environmental impacts due to the scale and regularity of meal production. Systematic life cycle assessments show that different food categories have markedly different greenhouse gas emissions, with ruminant meat and other animal-derived foods generally exhibiting higher emissions than plant-based foods (17). Similarly, dietary analyses indicate that meat and other animal products contribute disproportionately to diet-related greenhouse gas emissions (18). Moreover, high levels of food waste further increase the environmental burden of institutional food systems (19). Therefore, aligning menus with healthy and sustainable eating recommendations and improving nutritional care in these institutions is crucial for both residents and environment (20).

Considering all these factors in menu planning, artificial intelligence (AI) can be an effective tool to support menu planning by integrating nutritional, environmental, and operational constraints (21, 22). However, studies on the use of AI in the field of menu planning are limited in literature, and these studies generally include the evaluation of school menus (23, 24). In contrast, nursing homes represent a methodologically distinct and under-researched context, as residents typically rely almost entirely on provided meals, have limited dietary choice, and are exposed to the same menu structures over extended periods (12, 25). These characteristics can make menu planning in nursing homes particularly sensitive to both nutritional adequacy and sustainability considerations and can limit the direct transferability of findings from other studies. Therefore, this study aimed to comprehensively evaluate 12-week dinner menus (standard menus) and menus created using an artificial intelligence-based menu planning tool (optimized menus) delivered to nursing homes by a catering company in Eastern Norway in terms of energy and nutrient content, diet quality, and environmental impact (carbon and water footprints).

## 2 Materials and methods

This is an optimization study using existing nursing home menus provided by a catering company in Eastern Norway and does not involve observed dietary intake or intervention data.

In this study, menu planning principles and dish classification were informed by the catering provider’s established practices in nursing home menu planning, reflecting routine operational procedures in long-term care settings. In contrast, nutritional targets including total energy, nutrient content, and the percentage distribution of energy from macronutrients were defined according to guidelines issued by the Norwegian Directorate of Health (26).

### 2.1 Definition of nursing home menus

The catering company offers dinner menus to a total of 24 nursing homes and 2522 residents. There are two types of menus served in these nursing homes: (i) type 1: main dish + side dish + dessert or (ii) type 2: soup + main dish + side dish (Supplementary Table 1). In this study, “main dish” refers to dishes containing animal-based protein sources such as meat, poultry, or fish, whereas “side dish” refers to vegetable-based dishes served alongside the main dish. In addition, the Norwegian Directorate of Health recommends two different standard diets in institutions such as nursing homes: (i) a key advice diet and (ii) an energy- and nutrient-dense diet. The key advice diet is recommended for all healthy and ill people with good nutritional status, while an energy- and nutrient-dense diet is intended for people with poor appetite. This study was based on energy- and nutrient-dense menus, considering the high prevalence of malnutrition among nursing home residents reported in the literature.

### 2.2 Definition of energy- and nutrient-dense diet

The aim of the energy- and nutrient-dense diet is to prevent and treat malnutrition. This diet is recommended as a standard diet for nursing homes, home care, and daycare centers for older adults, as well as in some hospital departments (17). The diet consists of foods and dishes with a higher fat and protein content than that recommended in the key advice diet; therefore, the portion sizes are smaller.

In this diet, carbohydrate intake should provide 40–50% of total energy intake. Proteins should account for 15–20% of total energy intake. This corresponds to a protein intake of 1–1.5 g of good-quality protein per kg/bw. Protein quality is primarily determined by the protein’s content of essential amino acids. Foods that contain good-quality protein are primarily animal products such as fish, eggs, meat, milk, and dairy products. Fat in this diet should constitute 35–40% of total energy intake.

### 2.3 Dataset collection

Firstly, the dishes and their recipes in the food services dataset were collected and recorded on an Excel sheet. After that, the dishes were classified according to the dish types in Norwegian cuisine and based on the nursing homes’ menu planning principles, and a total of four dish types (soup, main dishes, side dishes, and desserts) were created. Finally, a total of 306 recipes were collected, including 14 soups, 58 main dishes, 199 side dishes, and 35 desserts. Then, the energy and nutrient contents, as well as the carbon and water footprints, of one portion of each dish were calculated.

The primary unit of analysis in this study was a 4-week dinner menu cycle. Three consecutive 4-week menus (12 weeks in total) were analyzed separately for both standard and optimized menus. In addition, an aggregated analysis was conducted across the full 12-week menu to provide an overall assessment of nutritional quality and environmental impact.

#### 2.3.1 Calculating the energy and nutrient content of dishes

The dishes’ energy, macronutrients (protein, carbohydrates, and fat), dietary fiber, vitamin B_12_ calcium, and iron content were calculated using the Norwegian Food Composition Table (18) according to their standard food recipes. After the calculation, the energy and nutrient contents of the optimized menus and standard menus were compared with recommendations of the Norwegian Directorate of Health (26).

#### 2.3.2 Calculating the carbon footprint of dishes

In the current study, Life Cycle Assessment (LCA) was employed to calculate the environmental footprint of various dish types. LCA is a holistic and globally standardized approach for examining the environmental effects of a product or system across its entire life cycle, from production and distribution to consumption and waste management (27). Carbon footprint values were obtained from two different databases relevant to the Nordic context: the Big Climate Database, developed by the Danish Consumer Council, and the Center for International Climate Research (CICERO) Database (28, 29). These sources offer average life cycle impact factors (kg CO_2_-eq) for commonly consumed food items in the region, based on ISO 14040/44-compliant LCA studies.

For the carbon footprint calculation, the amount of food in a portion of the recipes was determined, then the CO_2_ emissions per portion of each dish were calculated using the carbon footprint factors in the databases. Ingredients weighing less than 1% of the total weight of each recipe (e.g., spices, salt, garlic, etc.) were not included in the carbon footprint calculation (30). The results were expressed as kg CO_2_-eq.

#### 2.3.3 Calculating the water footprint of dishes

Water footprint values for each food item were obtained from the Water Footprint Network (WFN), which provides global average estimates of water use expressed in cubic meters per ton (m^3^/ton) (31, 32). These estimates are derived from a comprehensive LCA approach that considers blue, green, and grey water components. In this study, the total water footprint was utilized for each food item.

First, standard recipes for one portion of the dishes were determined. After that, water footprints were calculated using the m^3^/ton per product for each food using the water footprint factors. Ingredients weighing less than 1% of the total weight of each recipe (e.g., spices, salt, garlic, etc.) were not included in the water footprint calculation (30).

### 2.4 Evolutionary Automated Meal Generation (EvoMeal) software

*Evolutionary Automated Meal Generation (EvoMeal^1^)* is an open-source software first developed in Türkiye. *EvoMeal* enables the production of specially designed menus for different institutions. The tool optimizes menus by rearranging meal combinations without changing recipes, portion sizes, or nutritional content. It doesn’t directly alter energy and nutrient levels or food groups but improves overall menu balance. This makes EvoMeal easy to use in practice, as organizations can reduce their environmental impact without altering their existing meals or food service routines. The details of the software are given in detail in our previous study (33).

#### 2.4.1 Optimization algorithms

In our previous study, we used four different multi-objective optimization algorithms in the EvoMeal: (i) Non-dominated Sorting Genetic Algorithm (NSGA2), (ii) Reference-point-based Many Objective Evolutionary Algorithm (NSGA3), (iii) Multi-objective Selection Based on Dominated Hypervolume (SMSEMOA), and (iv) Adaptive Geometry Estimation-Based MOEA (AGEMOEA). Our primary findings showed that AGEMOEA and SMSEMOA overall outperformed both NSGA2 and NSGA3 (33). Therefore, based on previous results, we decided to use AGEMOEA in the current study.

In the current study, five criteria, each with the same level of importance, were defined to solve the menu planning problem: (i) *Energy, macronutrients (carbohydrates, protein, fat) and dietary fiber*, (ii) *Micronutrients (vitamin B_12_, calcium and iron):* The energy and nutrients that nursing home residents should take at dinner according to the recommendations of the Norwegian Health Directorate are summarized in Table 1. (iii) *Main ingredients:* The main ingredient in the daily menu should vary (For example: when fish soup is served, fish should not be included as the main dish). In addition, the same main ingredient should not be repeated on consecutive days (e.g., if fish is served on Monday, it should not be served on Tuesday). (iv) *Repetition:* Menus should be planned so that all dishes within the same week are different. (v) *Sustainability (carbon and water footprint):* Menus should be set to have as low a carbon and water footprint as possible.

**TABLE 1:** Optimization criteria used in menu planning.

Optimization criteria	Total	For dinner	Values
Energy (kcal)	1,900	30% of the energy ( ± 20%)	570 (456–684)
Carbohydrates (g)	40–50% of the energy	40–50% of the energy	64.13 (57.00–71.25)
Protein (g)	15–20% of the energy	15–20% of the energy	24.94 (21.38–28.50)
Fat (g)	35–40% of the energy	35–40% of the energy	23.75 (22.17–25.33)
Dietary fiber (g)	25–35	7.5–10.5	9.0 (7.50–10.50)
B_12_ (mcg)	4	1.2 ( ± 20%)	1.2 (0.96–1.44)
Calcium (mg)	950	285 ( ± 20%)	285.0 (228.00–342.00)
Iron (mg)	9	2.7 ( ± 20%)	
C footprint (CO_2_ eq)	As low as possible		
Water footprint (m^3^/ton)	As low as possible		

Based on these criteria, EvoMeal created a 12-week dinner menu (Supplementary Table 2).

### 2.5 Evaluation of the menu quality

Menu quality was evaluated using the Elderly-Nutrient Rich Food (E-NRF) 7.3 Index (34) and Dietary Diversity Score (DDS) (35).

#### 2.5.1 Elderly-nutrient rich food index 7.3 (E-NRF 7.3)

The E-NRF 7.3 score is based on a selection of nutrients relevant for older adults. Nutrients to encourage (NR7) include protein, dietary fiber, folate, vitamin D, calcium, magnesium, and potassium. Nutrients to limit (LIM3) comprise saturated fat, sodium, and total mono- and disaccharides. First, the NR7 and LIM3 scores were calculated for each menu per 100 kcal. Next, the individual LIM3 scores were subtracted from the NR7 scores, resulting in the E-NRF7.3 score. Higher E-NRF7.3 scores indicated higher nutrient density on a 100-kcal basis (34).

#### 2.5.2 Dietary diversity score

Dietary diversity is one dimension of diet quality. To calculate the diet diversity score (DDS), the foods consumed were classified into nine food groups recommended by the Food and Agriculture Organization (FAO): (1) cereals, roots, and tubers; (2) dark green leafy vegetables and vitamin-A-rich sources; (3) other fruits; (4) other vegetables; (5) legumes, nuts, and seeds; (6) meats; (7) oils and fats; (8) dairy products; and (9) eggs. Based on the consumption of these groups, scoring was made, and dietary diversity was assessed. Consumption of at least 15 g from any given food group was assigned a score of 1, whereas consumption of less than 15 g was assigned a score of 0 (35).

### 2.6 Statistical analysis

Statistical Package for Social Sciences software (SPSS, Version 23.0, United States, IBM Corp., 2015) was used for statistical analysis. Data visualization was performed with GraphPad Prism software (Version 8.0, San Diego, CA, United States) and R (Version 4.4.1). Normality was assessed using the Kolmogorov-Smirnov test. Data were expressed as mean ± standard deviation (X̄ ± SD) and min-max. The Independent Samples *t*-test was used to compare groups, and for all statistical analyses, *p* < 0.05 values were considered statistically significant.

## 3 Results

The comparison of energy and nutrient contents of the 4-week standard and optimized menus with the Norwegian Food-Based Dietary Guidelines (FBDG) is presented in Table 2. Across all three 4-week menus, the carbohydrate, dietary fiber, and calcium contents of the standard menus were within the recommended ranges. In contrast, the mean energy, protein, fat, vitamin B_12_, and iron contents of the standard menus consistently exceeded the upper limits of the recommended ranges.

**TABLE 2:** Comparison of energy and nutrient contents of 4-week standard and optimized menus with Norwegian Food-Based Dietary Guidelines recommendations.

Energy and nutrients	FBDG recommendations (mean, min-max)	Standard menus	p_1_*	Optimized menus	p_2_*
1st 4-week menu					
Energy (kcal)	570 (456–684)	737.15 ± 139.58	**< 0.001**	583.34 ± 50.94	0.172
Carbohydrates (g)	64.13 (57.00–71.25)	63.40 ± 16.45	0.468	57.52 ± 8.69	**< 0.001**
Protein (g)	24.94 (21.38–28.50)	42.09 ± 16.63	**< 0.001**	27.03 ± 5.65	**< 0.001**
Fat (g)	23.75 (22.17–25.33)	32.79 ± 14.59	**0.002**	22.32 ± 3.01	**0.005**
Dietary fiber (g)	9.0 (7.50–10.50)	10.09 ± 4.38	0.195	10.21 ± 2.12	**0.005**
Vitamin B_12_ (mcg)	1.2 (0.96–1.44)	4.23 ± 4.62	**0.001**	1.41 ± 0.23	**< 0.001**
Calcium (mg)	285.0 (228.00–342.00)	229.73 ± 125.38	**0.023**	241.10 ± 59.16	**0.001**
Iron (mg)	2.7 (2.16–3.24)	4.16 ± 1.82	**< 0.001**	2.81 ± 0.27	**0.049**
2nd 4-week menu					
Energy (kcal)	570 (456–684)	743.10 ± 140.51	**< 0.001**	591.24 ± 49.30	**0.031**
Carbohydrates (g)	64.13 (57.00–71.25)	64.41 ± 16.14	0.286	57.82 ± 7.97	**< 0.001**
Protein (g)	24.94 (21.38–28.50)	41.34 ± 16.20	**< 0.001**	23.90 ± 5.82	0.348
Fat (g)	23.75 (22.17–25.33)	33.33 ± 14.64	**0.001**	27.10 ± 5.14	**0.002**
Dietary fiber (g)	9.0 (7.50–10.50)	10.06 ± 4.82	0.248	10.04 ± 2.10	**0.015**
Vitamin B_12_ (mcg)	1.2 (0.96–1.44)	4.58 ± 4.30	**0.001**	1.34 ± 0.25	**0.007**
Calcium (mg)	285.0 (228.00–342.00)	251.87 ± 120.28	0.151	240.65 ± 67.00	**0.002**
Iron (mg)	2.7 (2.16–3.24)	4.03 ± 1.63	**< 0.001**	2.84 ± 0.38	0.066
3rd 4-week menu					
Energy (kcal)	570 (456–684)	747.28 ± 142.63	**< 0.001**	579.49 ± 52.67	0.345
Carbohydrates (g)	64.13 (57.00–71.25)	63.47 ± 16.35	0.451	56.82 ± 9.84	**0.001**
Protein (g)	24.94 (21.38–28.50)	41.96 ± 16.31	**< 0.001**	24.54 ± 5.57	0.736
Fat (g)	23.75 (22.17–25.33)	33.90 ± 14.96	**0.001**	25.93 ± 5.46	**0.043**
Dietary fiber (g)	9.0 (7.50–10.50)	10.34 ± 4.78	0.143	10.07 ± 1.64	**0.002**
Vitamin B_12_ (mcg)	1.2 (0.96–1.44)	4.08 ± 4.06	**0.001**	1.41 ± 0.25	**< 0.001**
Calcium (mg)	285.0 (228.00–342.00)	242.36 ± 116.92	0.059	237.76 ± 48.83	**< 0.001**
Iron (mg)	2.7 (2.16–3.24)	3.99 ± 1.65	**< 0.001**	2.90 ± 0.26	**< 0.001**

*Independent Samples *t*-test, *p* < 0.05. Data was given as mean and standard deviation (X̄ ± SD). p_1_, Comparison of standard menus with the FBDG recommendations; p_2_, Comparison of optimized menus with the FBDG recommendations. FBDG, Food-Based Dietary Guidelines. Bold values indicate statistically significant differences (*p* < 0.05).

For the optimized menus, energy and nutrient levels were generally closer to the FBDG reference values across the full 12-week menu. Although several nutrients (carbohydrates, protein, fat, dietary fiber, vitamin B_12_, calcium, and iron) showed statistically significant differences when compared with the FBDG reference means, these differences largely reflected deviations within the recommended ranges rather than non-compliance with the guidelines. The only exception was observed during the second 4-week menus, in which the mean fat content of the optimized menus slightly exceeded the recommended upper limit (Table 2).

**FIGURE 1:**
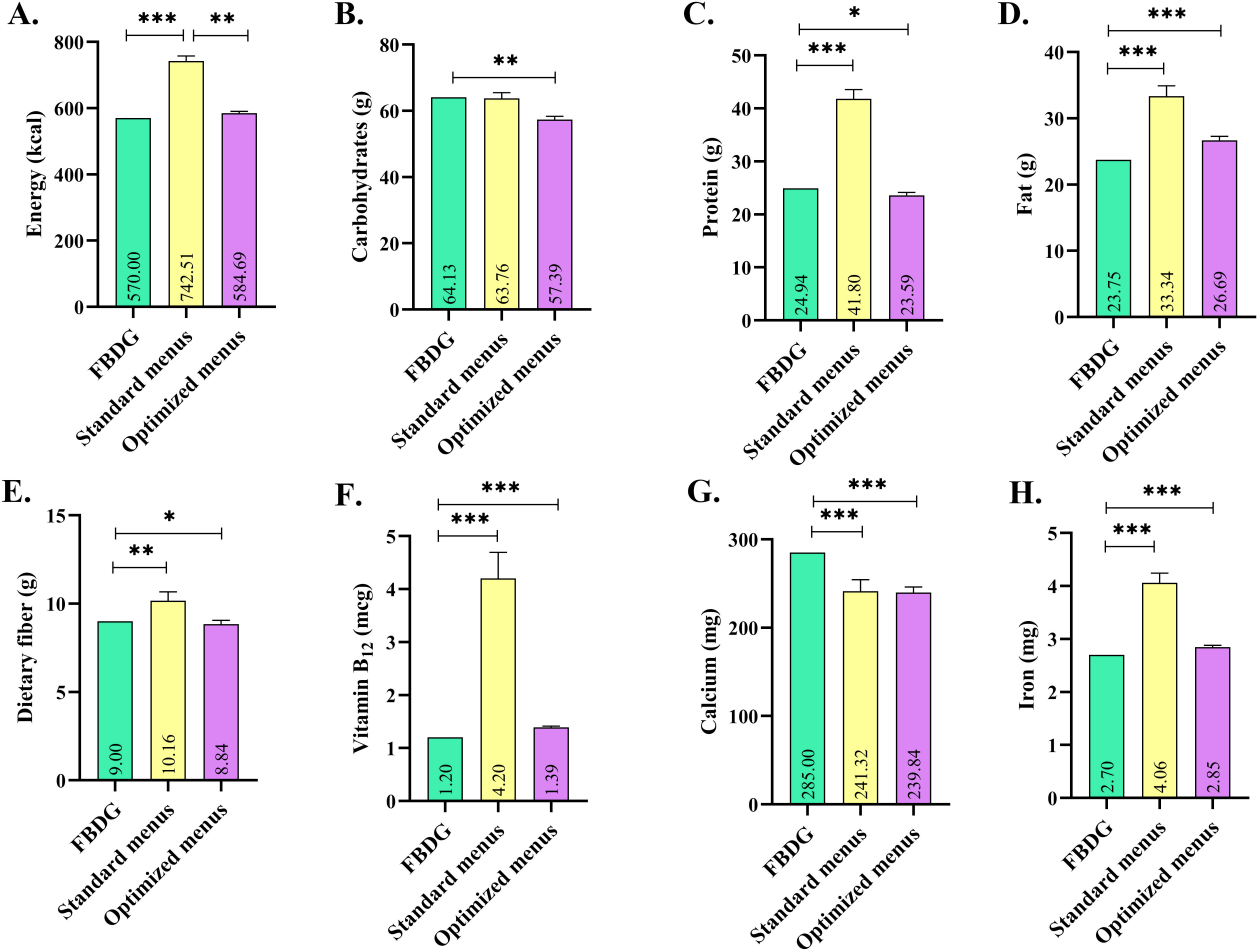
Comparison of energy and nutrient contents of 12-week standard and optimized menus with Norwegian Food-Based Dietary Guidelines recommendations. Data are given as mean and standard deviation (X̄ ± SD). Independent Samples *t*-test, *p < 0.05. ^**^p < 0.01, ^***^p < 0.001. FBDG, Food-Based Dietary Guidelines. **(A)** Energy (kcal). **(B)** Carbohydrates (g). **(C)** Protein (g). **(D)** Fat (g). **(E)** Dietary fiber (g). **(F)** Vitamin B12 (μg). **(G)** Calcium (mg). **(H)** Iron (mg).

Figure 1 summarizes the comparison of energy and nutrient contents of 12-week standard and optimized menus with Norwegian FBDG recommendations. In the standard menus, energy, protein, fat, vitamin B_12_, and iron levels were significantly higher than the recommendations (*p* < 0.001), while carbohydrate content was consistent with the guidelines (*p* > 0.05). Although dietary fiber was statistically higher and calcium was statistically lower than the FBDG mean values, both nutrients remained within the recommended range. Similarly, the optimized menus showed statistically significant differences from the FBDG values for most nutrients; however, all energy and nutrient contents were within the recommended ranges, indicating overall compliance with the guidelines.

**FIGURE 2:**
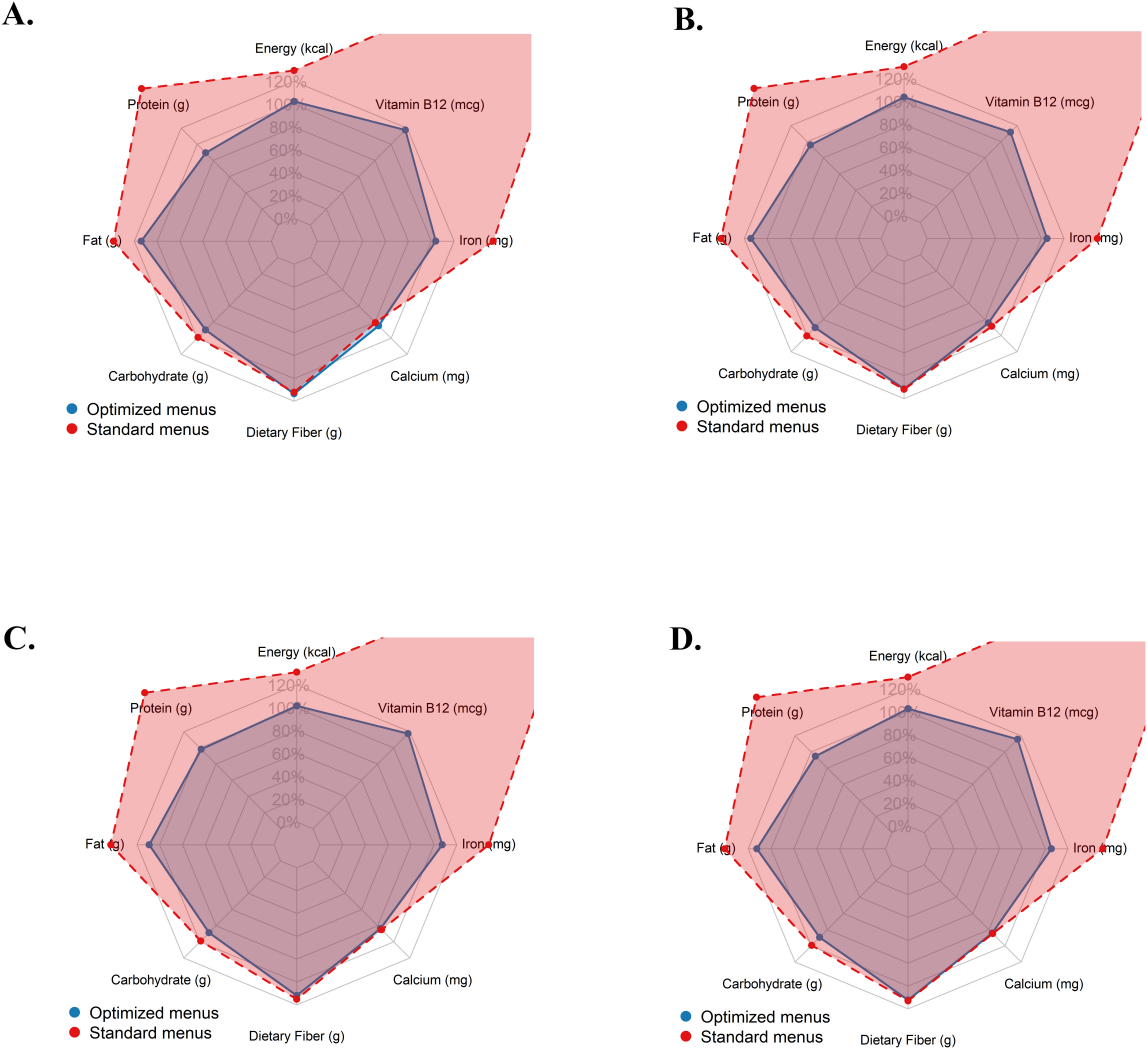
Radar plots of nutrient intake adequacy for standard and optimized menus based on the Norwegian Food-Based Dietary Guidelines. **(A)** 1^st^ 4-week menu. **(B)** 2^nd^ 4-week menu. **(C)** 3^rd^ 4-week menu. **(D)** 12-week menu.

Figure 2 presents the radar plots of nutrient intake adequacy for standard and optimized menus based on the Norwegian FBDG. According to these radar plots, standard menus had higher percentages of energy, protein, fat, vitamin B12, and iron across all periods. On the contrary, optimized menus consistently met nearly 100% of the requirements for all nutrients and were more consistent with recommendations. These findings supported the findings presented in Figure 1 and Table 2.

The comparison of E-NRF 7.3 scores, DDS, and environmental impact between standard and optimized 4-week menus is given in Table 3. E-NRF 7.3 scores and DDSs of the standard and optimized menus were found to be similar. Optimization of the standard menus resulted in E-NRF 7.3 changes ranging from −2.15 to +4.96%, and DDS changes from −0.70 to +3.74%. The carbon and water footprints of the optimized menus were significantly lower than those of the standard menus in the first and second 4-week menus. Although not statistically significant, a similar decreasing trend was also observed in the third 4-week menus. In addition, optimization of the standard menus resulted in carbon footprint changes ranging from −36.44 to −42.34%, and water footprint changes from −29.37 to −36.12%.

**TABLE 3:** Comparison of E-NRF 7.3 scores, Dietary Diversity Scores (DDS) and environmental impact between standard and optimized 4-week menus.

Parameters	1^st^ 4-week menu			2^nd^ 4-week menu			3^rd^ 4-week menu		
	Standard menus	Optimized menus	p*	Standard menus	Optimized menus	*p*	Standard menus	Optimized menus	*p*
E-NRF 7.3	13.46 ± 7.84	13.17 ± 5.10	0.872	13.11 ± 7.75	13.28 ± 5.13	0.924	13.30 ± 8.14	13.96 ± 5.26	0.722
*E-NRF 7.3 change (%)*	*−2.15*			*+1.30*			*+4.96*		
*DDS*	5.68 ± 0.77	5.64 ± 1.28	0.900	6.07 ± 1.05	5.64 ± 1.19	0.160	5.61 ± 1.20	5.82 ± 1.19	0.504
*DDS change (%)*	*−0.70*			*−7.08*			*+3.74*		
Carbon footprint (kg CO_2_ eq)	2.36 ± 2.23	1.39 ± 0.28	**0.026**	2.48 ± 1.14	1.43 ± 0.79	**0.024**	2.36 ± 2.20	1.50 ± 0.75	0.055
*Carbon footprint change (%)*	*−41.10*			*−42.34*			*−36.44*		
Water footprint (m^3^/ton)	1283.33 ± 877.01	819.75 ± 314.41	**0.011**	1337.51 ± 866.18	885.46 ± 455.77	**0.018**	1299.81 ± 926.07	918.00 ± 473.92	0.057
*Water footprint change (%)*	*−36.12*			*−33.79*			*−29.37*		

Data was given as mean and standard deviation (X̄ ± SD). *Independent Samples *T*-Test, *p* < 0.05. Italicized values indicate the change (%) between standard menus and optimized menus. Bold values indicate statistically significant differences (*p* < 0.05).

**FIGURE 3:**
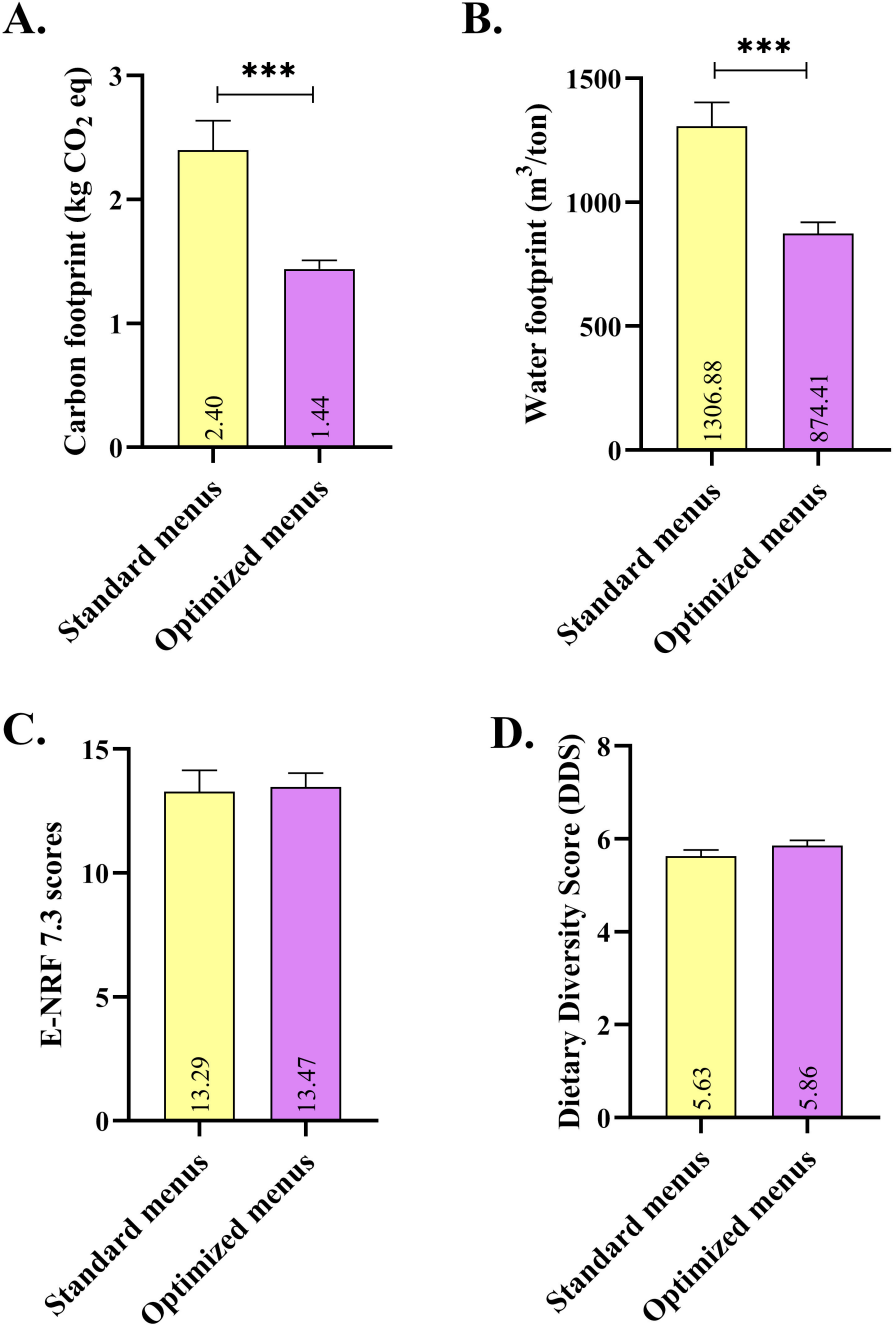
Comparison of E-NRF 7.3 scores, Dietary Diversity Scores, and environmental impact between standard and optimized 12-week menus. Data are given as mean and standard deviation (X̄ ± SD). Independent Samples *t*-test, *p* < 0.05. ^***^p < 0.001. E-NRF, Elderly-Nutrient Rich Food. **(A)** Carbon footprint (kg CO_2_ eq). **(B)** Water footprint (m^3^/ton). **(C)** E-NRF 7.3 scores. **(D)** Dietary Diversity Score (DDS).

Figure 3 demonstrates the comparison of E-NRF 7.3 scores, DDS, and environmental impact between standard and optimized 12-week menus. E-NRF 7.3 scores and DDSs of the standard and optimized menus were found to be similar. However, the carbon and water footprints of the optimized menus were significantly lower than those of the standard menus in the 12-week menus. In addition, optimization of the 12-week standard menus resulted in a carbon footprint reduction of −40.00%, and a water footprint reduction of −33.09% (Data not shown).

**FIGURE 4:**
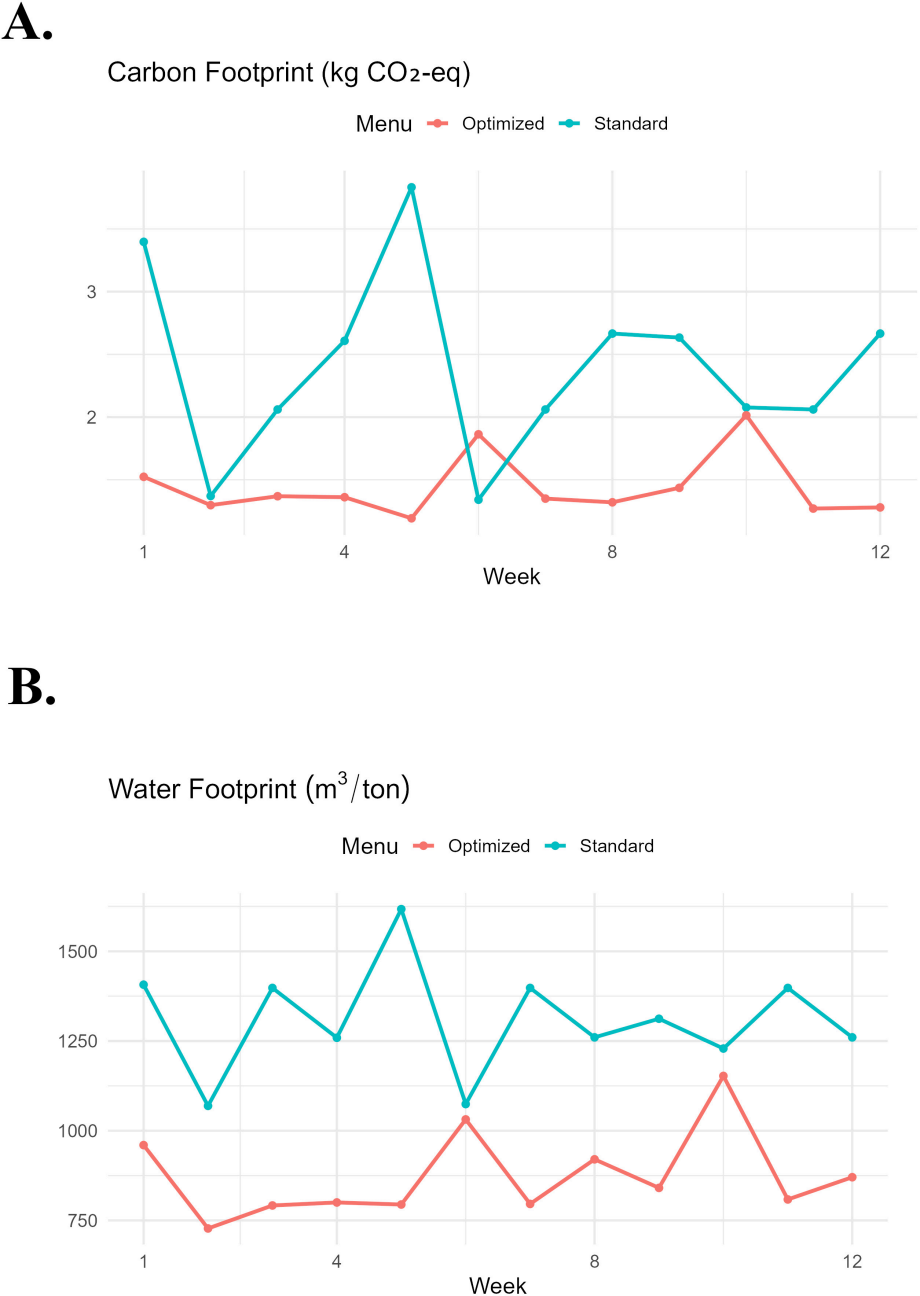
Weekly changes in carbon **(A)** and water footprint **(B)** of standard and optimized menus across 12 weeks.

Weekly changes in the carbon and water footprint of standard and optimized menus are summarized in Figure 4. The most significant differences in carbon footprint were observed during the 1st and 5th weeks. During these times, the standard menus exhibited peaks above 3.0 kg CO_2_-eq, while the optimized menus remained below 2.0 kg CO_2_-eq. A similar pattern was seen in the water footprint, with the most significant divergence occurring in the 5th week. The standard menus reached approximately 1,600 m^3^/ton, compared to around 850 m^3^/ton for the optimized menus this week. Despite weekly fluctuations, the environmental impact of the optimized menus consistently remained lower throughout the entire 12-week menu, except for the carbon footprint of the 5th-week optimized menu.

## 4 Discussion

Transformation of food systems is one of the most important issues for achieving public health and environmental sustainability (15). Previous studies on menu optimization in food services have generally focused on schools, universities or hospitals, rather than nursing homes specifically (15, 36–38). Similarly, OPTIMAT studies conducted in Sweden reported that school lunch menus developed through linear optimization reduced greenhouse gas emissions by 28–40% while maintaining nutritional composition consistent with Nordic Dietary Guidelines (24, 39, 40). To our knowledge, no study has comprehensively focused on optimizing nursing home menus in terms of nutritional composition, menu quality, and environmental impact. Furthermore, as far as we know, this study provides the first evidence from Norway on the comprehensive effects of menu optimization from food services. Therefore, current study may fill a significant gap by applying the EvoMeal to 12-week nursing home dinner menus and comparing standard and optimized menus in terms of nutritional composition, menu quality, and environmental sustainability. The study’s key findings demonstrate that AI-based menu optimization can increase compliance with the Norwegian FBDG without compromising menu quality, while simultaneously reducing environmental impacts in the nursing homes. These results suggest that AI could be an effective support for professionals in transforming food systems.

### 4.1 Comparison of nutritional composition of standard and optimized menus

The current study compared the energy and nutrient composition of standard dinner menus served in Norwegian nursing homes with optimized menus. As shown in Table 2 and Figure 1, optimization significantly improves the nutritional composition of the menus. Overall, optimized menus were more consistent with the FBDG recommendations, while standard menus exceeded the recommended upper limits for energy, protein, fat, vitamin B_12_, and iron at 4 and 12 weeks. Results from radar charts also supported this trend (Figure 2). While basal metabolism and physical activity decline with aging, high energy intake can lead to positive energy balance, resulting in unintended weight gain, obesity, insulin resistance, and increased cardiometabolic risk (41). Similarly, chronically excessive protein intake can place additional physiological stress on the kidneys, particularly contributing to age-related decline in kidney function (42). High-fat consumption, particularly saturated fat, may increase the risk of some cardiovascular events (43). Furthermore, the high iron and vitamin B_12_ content of standard menus largely reflects the consumption of high-bioavailable red meat. However, long-term consumption of high amounts of red meat is associated with adverse outcomes, and an increased risk of type 2 diabetes and cardiovascular disease (44, 45). Epidemiological data also suggest that red and processed meat consumption increases the risk of colorectal cancer through heme iron and nitrous compounds (46). Therefore, while red meat is nutritious, its consistent high consumption may pose a risk to metabolic and gastrointestinal health. Given that older people are more prone to malnutrition due to physiological changes, chronic diseases, and decreased appetite, balanced menus are crucial (47). In this regard, considering the nutritional composition of optimized menus, EvoMeal could be a promising planning tool for nursing homes. In fact, studies conducted across institutions, such as hospitals and schools, show that various optimization methods improve the nutritional composition of menus (15, 36).

### 4.2 Comparison of nutritional quality of standard and optimized menus

In this study, the E-NRF 7.3 score and DDS were used to assess menu quality. As shown in Table 3, the E-NRF 7.3 values of the optimized and standard menus ranged from 13.11 to 13.96, and the DDS values ranged from 5.64 to 6.07. Over the 12-week menu evaluation, the average E-NRF 7.3 score for the optimized menus was 13.47 and 13.29 for the standard menus. The average DDS was 5.86 for the optimized menus and 5.63 for the standard menus (Figure 3). These results indicate that there were no statistically significant differences between the optimized and standard menus in terms of E-NRF 7.3 and DDS. The combined evaluation of nutrient density (E-NRF 7.3) and dietary diversity (DDS) suggests that the dinner menus served in nursing homes generally offer a good nutritional profile. Mean E-NRF 7.3 scores of approximately 13 are consistent with the 10–15 range reported in validation studies in older individuals in Europe; moreover, higher scores, including above 10, have been associated with more favorable micronutrient status (34, 48). The mean DDS of 5.50–6.00 indicates a near-moderate level of variety at evening meals, which is quite close to the threshold of ≥ 6 generally used for moderate variety. This variation is important because previous studies have reported that greater diet diversity is associated with lower frailty and lower all-cause mortality in older adults (49, 50). The relatively moderate DDS values in our study are due to the evaluation of dinner menus only; it is important to consider that the overall level of variety is likely higher when other meals of the day are included. Previous study in Iran where university menus were optimized using linear programming method reported significantly higher NRF indices (15). The similarity between the DDS and NRF 7.3 contents of the optimized and standard menus in our study suggests that comprehensive nutrition planning is already implemented in Norwegian nursing homes; therefore, the opportunity for further improvements in diet quality in the optimized menus may be limited.

### 4.3 Comparison of environmental impact of standard and optimized menus

Optimizing menu combinations using EvoMeal without changing existing meal components resulted in a significant reduction in the carbon and water footprint of menus (Figure 3 and Table 3). This finding demonstrates that environmental impact depends not only on the nutrients used but also on how meals are assembled. The literature also emphasizes that even small changes can be significant in reducing overall environmental impact (18, 51). This approach offers the potential to increase sustainability without altering the nutritional content of meals served in nursing homes, providing institutions with a feasible, economical, and acceptable improvement path. Furthermore, the findings suggest that facilitating the use of digital optimization tools could make sustainable menu planning more accessible in nursing homes. Our results are consistent with previous studies demonstrating that different optimization methods in menu planning are effective tools for sustainability (15, 36).

### 4.4 Trade-offs between sustainability and clinical nutrition in menu planning in nursing homes

An inherent challenge in sustainability-oriented menu optimization is balancing environmental objectives with clinical nutrition priorities, particularly in geriatric care. Previous studies have highlighted that efforts to reduce the environmental footprint of diets may introduce trade-offs with nutritional adequacy if not carefully managed (52, 53). In long-term care settings, maintaining adequate energy and nutrient intake is a central component of preventing malnutrition and supporting healthy aging (47). While the present study demonstrates that environmental impacts can be reduced without altering menu content or compromising guideline-based nutritional adequacy at the menu level, it does not assess whether such changes translate into maintained intake at the individual level. This underscores the importance of evaluating both sustainability performance and nutritional outcomes when applying optimization models in institutional food services.

### 4.5 Limitations of the study

The current study has some limitations. First, the menu evaluation was based on planned menus and did not include data on actual food consumption, plate leftovers, or residents’ acceptability of the meals, which limits conclusions about real-world dietary intake and food waste. In this context, the lack of validation through sensory analysis and intake monitoring represents an additional limitation, as it remains unclear whether sustainability gains achieved through optimization are accompanied by maintained energy and nutrient intake at the resident level. Second, the optimization models rely on assumptions embedded in nutritional databases and environmental sustainability indicators, which may influence the estimated outcomes. In addition, economic aspects such as food costs, cost-effectiveness, and potential budgetary implications were not assessed. Finally, operational factors including staff workload, food preparation practices, and purchasing processes were beyond the scope of this analysis. Therefore, future studies should test optimized menus under real-life conditions, integrating sensory evaluation, intake monitoring, consumption data, plate waste, acceptability, and economic performance to better capture the practical implications of menu optimization in nursing home settings.

## 5 Conclusion

In conclusion, this study demonstrates that menus optimized with EvoMeal significantly improve compliance with the Norwegian FBDG and offer a feasible approach for aligning the nutrition of older adults with sustainability goals. The results indicate that digital optimization tools can support nutritionally adequate and environmentally sustainable menu structures in nursing home settings.

Beyond operational aspects, this study contributes to public health by showing how institutional menu planning can address sustainability concerns while preserving nutritional quality in long-term care. The findings further suggest that environmental impacts can be reduced through menu reorganization without modifying existing menu content or compromising guideline-based nutritional adequacy. This low-barrier approach may help maintain continuity in food provision and limit disruption to routine care practices.

Overall, the study suggests that sustainability-oriented menu optimization in nursing homes can be implemented without evident trade-offs in nutritional quality. However, future research is needed to evaluate consumption, acceptability, and health-related outcomes in real-life settings.

## Data Availability

The original contributions presented in this study are included in the article/Supplementary material, further inquiries can be directed to the corresponding author.

## Footnotes

1
https://github.com/omursahin/EvoMeal
