# Supplementary material for: Optimizing nursing home menus in Norway from a sustainability and nutritional perspective
Source: Front Nutr. 2026 Apr 16;13:1776523. doi: 10.3389/fnut.2026.1776523 (PMC13128410; doi:10.3389/fnut.2026.1776523)
Supplement: Supplementary file 1 [file Table_1.docx]

**Supplementary Table S1.** Examples of menu types based on institutional serving structure in Norwegian nursing homes

| **MENU TYPE 1** | **EXAMPLE 1** | **MENU TYPE 2** | **EXAMPLE 2** |
| --- | --- | --- | --- |
| **Main dish** | Norwegian meat patties with pepper sauce | **Soup** | Cauliflower and broccoli soup |
| **Side dish** | Boiled potatoes, Julienne root vegetable | **Main dish** | Chicken in curry |
| **Dessert** | Carrot cake | **Side dish** | Boiled potatoes, Julienne root vegetable |

There are two types of menus served in these nursing homes: (i) type 1: main dish + side dish + dessert or (ii) type 2: soup + main dish + side dish. In this study, ‘main dish’ refers to dishes containing animal-based protein sources such as meat, poultry, or fish, whereas ‘side dish’ refers to vegetable-based dishes served alongside the main dish.
